# The ubiquitous role of mitochondria in Parkinson and other neurodegenerative diseases

**DOI:** 10.3934/Neuroscience.2020004

**Published:** 2020-03-25

**Authors:** Georgia Theocharopoulou

**Affiliations:** Department of Informatics, Ionian University, Plateia Tsirigoti 7, 49100, Corfu, Greece

**Keywords:** Parkinson' disease, Alzheimer's disease, neurodegeneration, mitochondria dynamics, protein aggregation, fusion, fission

## Abstract

Orderly mitochondrial life cycle, plays a key role in the pathology of neurodegenerative diseases. Mitochondria are ubiquitous in neurons as they respond to an ever-changing demand for energy supply. Mitochondria constantly change in shape and location, feature of their dynamic nature, which facilitates a quality control mechanism. Biological studies in mitochondria dynamics are unveiling the mechanisms of fission and fusion, which essentially arrange morphology and motility of these organelles. Control of mitochondrial network homeostasis is a critical factor for the proper function of neurons. Disease-related genes have been reported to be implicated in mitochondrial dysfunction. Increasing evidence implicate mitochondrial perturbation in neuronal diseases, such as AD, PD, HD, and ALS. The intricacy involved in neurodegenerative diseases and the dynamic nature of mitochondria point to the idea that, despite progress toward detecting the biology underlying mitochondrial disorders, its link to these diseases is difficult to be identified in the laboratory. Considering the need to model signaling pathways, both in spatial and temporal level, there is a challenge to use a multiscale modeling framework, which is essential for understanding the dynamics of a complex biological system. The use of computational models in order to represent both a qualitative and a quantitative structure of mitochondrial homeostasis, allows to perform simulation experiments so as to monitor the conformational changes, as well as the intersection of form and function.

## 1. Introduction

Mitochondria related diseases have been identified even from the premolecular era (1962–1988), when Milton Shy and Nickolas Gonatas have stated the value of mitochondrial DNA (mtDNA) regarding the studies of two myopathies which they have given the greek names pleoconial myopathy and megaconial myopathy [1].

Through the molecular era, mitochondria have been linked to several human disorders, which include mitochondrial failure in complex diseases, such as autism spectrum disorder (ASD), as well as defects in mitochondrial dynamics [2], [3]. Genetic studies of pathogenic mutations in mitochondrial proteins which participate in mitochondrial dynamics, have unveiled the critical role of mitchondrial dynamic changes in numerous neurological disorders [4], [5]. Mutations in the mitochondrial genome appear to cause defects in axonal transport, in central, as well as in peripheral nervous system, which are a common characteristic in optic atrophy, hereditary spastic paraplegia (SPG) and Charcot-Marie-Tooth disease (CMT) [6]. Many biological research studies support the link between altered mitochondrial dynamics and some of the most common neurodegenerative diseases, such as Parkinson's (PD), Alzheimer's (AD) and Huntington's (HD) disease [7]–[11].

Mitochondrial dynamics, a process which has a vital role in the cell, describe the continuous changes in shape of mitochondrion, in response to stimuli in order to meet functional requirements. Dynamics involve interactions on different levels of the molecular organization: overall morphology, shift and reorganization of mitochondrial proteins and DNA [12]. Mitochondrion is capable of fusing and budding, requiring essential protein complexes, which act as mediators. These processes of fission and fusion play a key role in the viability of mitochondria, since they are essential components of a complex system, representing a “clean-up” process of impaired mitochondrial elements [13].

In neuronal cells, mitochondria can be found enhanced at areas of increased energy demand. Unveiling the mechanisms of fission and fusion, which essentially regulate mitochondrial arrangement and motility increases understanding of trafficking and regulation of mitochondrial transport. Mitochondria alter their motility in axons and at synapses in order to maintain energy homeostasis that is essential for synaptic functions [14]. Defective mitochondrial transport is implicated in failure of axonal regeneration after injury and in various neurological disorders, including AD and PD [15].

Mitochondria-derived ATP production provides most of the axonal energy. Damaged mitochondria fail to produce ATP. Bioenergetic deficits and chronic oxidative stress trigger axonal pathology and synaptic dysfunction, thus contributing to pathogenesis of neurodegenerative diseases [16]. In addition to mitochondrial dynamics, their bioenergetic state has a key effector to homeostasis in order to modulate neuronal morphology and connectivity [17].

Here, we discuss the pathogenic role of abnormal function of mitochondria in the development of age-related neurodegeneration. The three aspects of mitochondrial function: (1) dynamics, i.e. fusion and fission, (2) kinetics, i.e. transport and distribution and (3) bioenergetics of the electron transport chain and the TCA/Kerbs cycle, are tightly regulated and associated with disease states when mutations arise. Biological studies from research in animal model systems, present findings confirming the hypothesis that, mitochondial dysfunction underlie the progression of impaired neuronal function in major neurodegenerative disorders. Mitochondria have a key role supporting the anti-ageing cell repair mechanisms. In order to elucidate the complex interactions in mitochondrial quality control, we highlight the use of computational modelling, for analysing the functional role of these organelles, implicated in neurodegeneration.

## 2. The critical role of mitochondria dynamics

As shown in several studies mitochondria adopt varying morphologies which can be cell type-/tissue-specific [18], [19]. Mitochondria form a dynamic network which transforms from long tubules to small round vesicles. Fusion and fission of mitochondria are complementing each other in order to achieve a dynamic organizational equilibrium. The mechanism that excites these two opposing processes depends on various elements, such as cell type and cellular metabolic requirements. Moreover, these processes are interdependent and act as protective mechanisms. Fusion provides the possibility to mitochondria to reorganize, thus allowing protein complementation and repair of mitochondrial DNA [7]. As mitochondrial fusion promotes the exchange of contents between mitochondria, deficient cells have a way to regain essential components. In the cerebellum, mitochondrial fusion is indispensable to cell viability and mitochondrial transport in neurons [20]. Regardless the role that fusion plays in mitochondrial function, it is required for the preservation of mitochondrial integrity. Mitochondrial fusion is succeeded by fission. In this way, mitochondrial contents reorganization is accomplished and damaged mitochondrial components are enriched [21].

Both outer and inner mitochondrial membrane fusion are mediated by GTP-hydrolyzing proteins. In mitochondrial fusion the key proteins required for the initial tethering in the outer membrane are mitofusins (Mfn1 and Mfn2) [22], [23]. The other mediator of mitochondrial fusion in mammals is Optic atrophy protein 1 (OPA1), which acts in the inner mitochondrial membrane [24]–[26]. Apart from fusing the inner membrane, OPA1 tethers mitochondrial cristae in order to maintain their shape. Defects in either, or both processes, have been associated to dominant optic atrophy, which is characterized by OPA1 mutations in humans [24], [27], [28]. The opposite function of mitochondrial fusion, fission, requires a dynamin related/like protein 1 (Drp1/Dlp1) [29], [30]. Mitochondrial division is critical for the remodelling and structural rearrangement of mitochondrial networks. In case of irregular fission, mitochondrial network displays very long and interconnected mitochondria. More, specifically, has been reported that loss of ChChd3, an inner mitochondrial membrane protein, caused mitochondria with lower crista density and more tubular crista [31]. Several research groups detected that a different gene product, called Fis1, is crucial for recruiting Drp1 to sites of scission [32], [33]. Also, mitochondrial fission factor (Mff), an adaptor molecule, interacts with hFis1 and stimulates polymerization via recruiting Drp1 [34]. Moreover, protein kinases that regulate phosphorylation of Drp1 also efficiently stimulate fission [12], [35]. Another mitochondrial protein that acts as a modifier of mitochondrial fission pathway is Ganlioside-induced differentiation-associated protein 1 (GDAP1) [36], [37].

The functional processes that control mitochondrial dynamics entail a number of factors, including coordination mechanisms, as well as cellular mechanisms, which act as response to extramitochondrial stimuli. Consequently, the resulting mitochondrial morphology depends on up and down regulation of these essential proteins. More specifically, reduction of Mfn1 induces a scattered network of small vesicular mitochondria, whereas knockout of Mfn2 produces larger vesicular mitochondria located around the nucleus [38], [12]. Furthermore, studies *in vivo* with Mfn2-deficient Purkinje cells showed disorders in mitochondrial distribution and electron transport chain activity, because of the inhibition of fusion events [20].

Deficient Drp1 function in neurons induces an elongated mitochondrial complex that expands to the neurites [30]. Studies in mice cerebellum Purkinje cells, showed that Drp1 depletion during embryonic brain development modified mitochondrial architecture from short tubules to large spheres [39].

Obviously, the dynamic nature of mitochondria facilitates a quality control mechanism, in order to advance protection against damaged organelles. Mitochondrial remodelling and key proteins involved in this process, have also been implicated to play a vital role in mitophagy and autophagy as a nonselective degradation system, highlighting their implication in the pathogenesis of neurodegenerative diseases and their potential as a target-driven therapeutic strategy [40]. For example, in a study in postmitotic neurons [41] findings suggest that Mfn1/2 dissociation from mitochondria is essential for mitophagy to proceed, while another study correlates OPA1 overexpression with reduced mitochondrial autophagy due to increased mitochondrial size [21]. In [42] Lutz, Exner, Fett, et al.(2009) suggest that, modification in both mitochondrial morphology and ATP production, due to either Parkin or PINK1 loss of function, could be preserved by the mitochondrial fusion and fission proteins (Mfn2, OPA1 and Drp1). Studies with Drp1 knockout in Purkinje cells resulted in altered mitochondrial morphology and accumulation of autophagy markers [41], indicating that completion of mitophagy requires both fusion and fission processes. In other studies scientists proposed a mechanism in which PINK1 and Parkin participate in targeting mitochondria for mitophagy in case of dissipated mitochondrial membrane potential [43], [44].

Although, several proteins have been implicated in the mitophagic process (i.e. Nix, Parkin, PINK1, and FUNDC1), the molecular signals to trigger mitophagy in mammalian cells and target mitochondria to autophagosomal membranes, are still being investigated. Although various techniques have been used to monitor mitophagy in neurons, where spatiotemporal alterations characterise mitochondrial network, understanding and quantification of mitophagy remains a great challenge [45].

## 3. Altered mitochondrial dynamics in neuronal diseases

**Figure 1.**
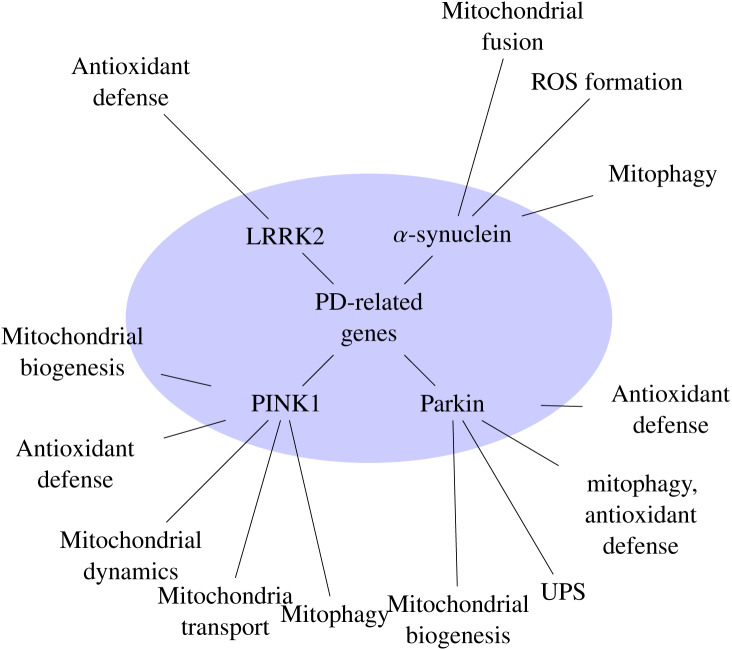
PD-related genes and their involvement in mitochondrial function.

In the last years many lines of evidence implicate disorders in mitochondrial dynamics to be associated with various neurodegenerative diseases, (refer to Table 1) [10], [46]–[49]. Parkinson's disease, is one of the most common degenerative disorders of the central nervous system, characterized by movement disorders, as well as cognitive decline. Motor symptoms are predominantly the result of degeneration or loss of dopaminergic neurons [50]. PD is predominantly a sporadic condition, for which more than 40 genetic risk-loci have been identified [51]. However, 5–10 % of patients have been reported with mutations in a range of genes inherited in an autosomal dominant pattern [52]. Studies investigating mitochondrial function and integrity, reported that Parkin acts downstream of PINK1, two common PD-linked genes [53]. Further studies have showed that Parkin is selectively recruited to the mitochondria and regulates critical mitochondrial remodeling processes [43], [54]. Recently, in [55], Billingsley et al. (2019), calculated a mitochondrial-specific polygenic risk score (PRS) and found that mitochondrial function-associated genes show a functional consequence correlated with PD risk. The PD-related genes have long been reported to be involved in mitochondrial homeostasis [56], [57] (refer to Figure 1).

Moreover, respiratory chain complex dysfunction has been associated with the pathogenesis of PD [58]. Alpha-synuclein has been associated in case of sporadic as well as familial forms of PD [59], [60]. Synuclein has been reported to have a primary effect on mitochondrial morphology [61]. Nevertheless, the autosomal recessive forms of PD produced by loss of Parkin and PINK1 differ clinically and pathologically from idiopathic PD [62]. In particular, most cases of recessive PD do not appear to involve the accumulation of synuclein [63].

Mutations in genes of fusion essential proteins, OPA1 and Mfn2, have been associated with two neurodegenerative diseases, autosomal dominant optic atrophy (ADOA) [27], [28], [64]–[66] and CMT type 2A [67]–[70], respectively. Patients with neuropathy, optic atrophy and other CNS disorders have been identified with Mfn2 mutations. In a recent study [71], the clinical phenotypes of patients with CMT2A have been investigated and it has been shown that Mfn2 mutation accounted for 91% of all patients. Still, the mechanisms by which Mfn2 mutants affect the function of peripheral nerve cells remain unclear. Investigation of the mutations that cause CMT2A, supports the hypothesis that, regulation of mitochondrial fusion events is crucial for the viability of neuron cells. Studies indicate that, in cells with lower expression of Mfn1, or when higher levels of mitochondrial fusion is required, such as in peripheral neurons, mutations in Mfn2 lead to axonal degeneration, characteristic in CMT2A neuropathy [72].

**Table 1.** Proteins reported to be involved in neurodegenerative diseases.

Phenotype/Disease	Gene product/Role in mitochondrial function	Induced dysfunction	References
PD	Pink1/Mitochondrial morhogenesis, mitophagy, mitochondrial fission-fusion, mitochondria transport in axon	Neurons degeneration accompanied with mitochondria- related structural alterations	[73]–[75]
PD	Parkin/Mitochondrial biogenesis, UPS, mitophagy, antioxidant defense	Impaired mitochondrial function and morphology, oxidative damage	[73],[76],[77]
PD	Alpha-synuclein/Mitophagy, ROS formation, mitochondrial fusion	Mitochondrial fragmentation, impaired respiratory complex	[78],[79]
PD	LRRK2/Regulating mitochondrial homeostasis	Reduced mitochondrial membrane potential and total intracellular ATP levels, mitochondrial elongation and interconnectivity	[80],[81]
CMT2A	Mfn2/fusion, transportation of mitochondria, axonal transport	Accumulations in mitochondrial network, Aberration in Δ*ψ*m, Disorders in mitochondrial fusion	[38],[71],[82],[83]
CMT4A	GDAP1/fission, regulation of mitochondria network	Elongated mitochondria, Oxidative stress, Neuronal demyelization	[36],[84],[85]
Abnormal brain development, Optic atrophy	Drp1/fission	Elongated mitochondria, fission disruption	[29],[30],[39],[86]
Dominant Optical Atrophy	OPA1/fusion	Fragmented mitochondria, Aberrations in ATP production, Δ*ψ*m and oxygen consumption, Instability of mtDNA, Sensitivity to apoptosis	[24],[27],[28],[64],[66],[87]

In a mouse model used in order to investigate the contribution of defective fusion in neurodegeneration, experimental results revealed that mutant purkinje cells have aberrant mitochondria distribution, lacking mitochondrial DNA nucleoids and electron transport activity [20]. Reduced fusion rate, given the role that plays in mitochondrial distribution, might cause dissipation in levels of ATP at the pre- and post-synaptic terminals and thus is essential for the long-term conservation of the nervous system.

Mutations in GDAP1, another protein involved in the regulation of mitochondrial network, has also been implicated in early-onset CMT [84], with a great heterogeneity of phenotypic presentations, from demyelinating, axonal or intermediate, suggesting that different molecular mechanisms may underlay the pathogenesis of the disease. Interestingly, in recent studies a new protective role has been proposed for GDAP1, identified in neuronal cell death associated with intracellular antioxidant glutathione (GSH) depletion [85]. Their results showed that over-expression of GDAP1 protected against oxidative stress caused by depletion of GSH that disturbs mitochondrial membrane integrity.

Several studies propose that mitochondrial dysfunction and oxidative damage play an important role in the pathogenesis of AD [88]–[90].

In addition to the characteristic amyloid plaques and neurofibrillary tangles, oxidative stress - induced mitochondrial dysfunction is one of the characteristic features in AD, as a potential pathogenic factor, affecting both neuronal and peripheral cells. A decade ago, research findings from *in situ* analysis of damaged brain tissue, reported increased mtDNA in the cytoplasm and a significant decrease in mitochondria [91]. Researchers postulate that, neurons in AD show increased mitochondrial degradation products, due to either turnover of mitochondria by autophagy, or a reduction of proteolytic turnover. Moreover, prior studies reported evidence that link oxidative stress to neuronal death and neural dysfunction [92]. Recently, it has been reported that there is a relationship between neuronal oxidative damage and mitochondrial abnormalities [93]. Interestingly, studies with AD patients and cultured fibroblasts, demonstrate how abnormalities in the regulation of mitochondrial fusion and division contribute to mitochondrial and synaptic dysfunction in AD [94]. From these studies' results, abnormal mitochondrial morphology and distribution, seem to be connected to decreased levels of Dlp1, which regulates mitochondrial fission. Raised levels of oxidative stress and increased amyloid *β* production are involved as pathogenic factors that cause Dlp1 reduction and aberrant mitochondrial allocation in AD cells [95].

The finding that mutations in genes involved in fusion and fission are connected with hereditary motor and sensory neuropathies, implies that balancing of these processes is critical for the myelinated peripheral nerves. It appears that oxidative stress and accumulation of misfolded proteins, impair mitochondria's dynamics in neurons, resulting not only in mitochondrial morphological changes and structural impairment, but also leading to mitochondrial re-distribution in the cell body towards the axon, or dendrites [8], [86].

These observations show that, proper regulation of mitochondrial dynamics is a critical factor for the proper function of neurons. Specifically, these findings suggest that, abnormal mitochondrial dynamics cause mitochondrial and neuronal dysfunction, including synaptic dysfunction and eventually neurodegeneration. Considering how much neurons depend on mitochondrial function, it should come as no surprise that disruption of the normal mitochondrial life cycle, plays a key role in the pathology of neurodegenerative diseases. Still, the relationship between mechanisms of mitochondrial disorders and neuronal degeneration, remains to be determined.

## 4. Mitochondria trafficking

Another critical aspect of mitochondrial dynamics is movement and axonal transport, which is essential for the distribution of functional mitochondria to distal synaptic terminals. The importance of this aspect is crucial in highly polarized neurons. Because of their highly polarized nature and their complex architecture, neurons demand high energy from mitochondria at sites distant from the cell body. Thus, although mitochondria's presence is ubiquitous in neurons, they are not randomly distributed, but they appear to concentrate in specific axonal regions that have a high demand for ATP.

Mitochondria move along microtubule tracks via the ATP-dependent motor proteins, kinesin and dynein [96], [97]. Moreover, they can be transported bidirectionally, via myosin motors along actin filaments [98], [99].

The motor proteins kinesin and dynein are mechanochemical enzymes, which operate by consuming energy, provided by the ATP hydrolysis, in order to move cellular cargo along neuronal axon. The microtubules transport of organelles such as mitochondria and vesicles containing neurotransmitters are mediated by the Kinesin ATPase activity towards microtubules' plus end [100]. In retrograde transport, another motor protein, Dynein, carries organelles and microtubule fragments along neuronal axon in reverse direction, towards the cell body [100]. Genetic studies indicate that axonal transport motor proteins may have biochemical and/or biophysical interdependency [101], [102].

Although proteins that are responsible for axonal transport have been identified, mechanisms that control mitochondrial trafficking have not been cleared yet. Studies have identified that GTPases Miro and Milton are required for mitochondrial transport, as linkers of mitochondria to motor proteins [103], [104]. Recent studies suggest that drosophila Miro affects mitochondrial transport, by controlling motor proteins and coordinating either antero- or retrograde mitochondrial movement [105]. Loss of Miro appears to cause reduction of mitochondria from dendrites and axons, resulting in neurotransmission defects. [104]. Moreover, mutations in linker protein Milton, result in reduction of mitochondria from the nerve terminal, while other organelles maintain their normal distribution [103]. Furthermore, deregulated interaction between mitochondria and the cytoskeleton during trafficking, may promote changes in mitochondrial morphology [106].

Studies reported that PD related genes regulate mitochondrial trafficking [107]. PINK1 and Parkin associate with motor/adaptor Miro on depolarized mitochondria. Their findings suggest that PINK1/Parkin pathway sequesters damaged mitochondria to their clearance.

Recently, in [108] Hsieh et al. (2016), reported functional impairment in Miro degradation and mitophagy both in familial and sporadic PD. Hsieh et al. (2016) found that Miro deficiency also causes mitochondrial autophagy defects in sporadic PD cases. Moreover, in familial PD, pathogenic LRRK2 disrupts Miro removal from damaged mitochondria and consequently delays the onset of mitophagy. Thus, degradation of mitochondrial motility and mitophagy appears to be a shared feature in familial and sporadic PD.

A growing number of studies focus on the interaction between mitochondrial motility and fusion/fission dynamics [109], which suggest that there are important functional connections between mitochondrial dynamics and axonal transport. These studies, include reports indicating that, mitofusins play an essential role in regulating mitochondrial transport. Experimental results show that Mfn2 mutant proteins alter microtubule-based transportation of mitochondria [69], [110]. This link has been made by the observation that Mfn2 disease mutants disrupt mitochondrial movement, but do not alter transport of other organelles.

Distributing mitochondria over long distances in order to satisfy an ever-changing demand for energy supply, is a challenging task that requires a controlling mechanism. Not only mitochondrial transport must be regulated, but also retention of a healthy population to sites of interest. When this machinery is compromised pathological consequences are induced, which may include axonal transport impairment, or even axonal loss.

## 5. Neurodegenerative diseases and axonal transport defects

Mutations in motor proteins have been identified to cause motor neuron degenration [111]. Further studies have shown that defects in axonal transport contribute to the pathology induced by mutations in other genes. Genetic analysis of long-distance axonal transport supports the hypothesis that interruption in axonal transport is implicated in a number of progressive neurodegenerative diseases including AD, PD, HD, Amyotrophic lateral sclerosis, etc. one common feature is, that genes linked to these diseases encode proteins transported in neuronal axon, e.g. preselin 1 and APP in AD, PINK1/Parkin in PD, Cu/Zn superoxide dismutase (SOD1) in ALS and huntingtin (Htt) in HD [112]–[114]. Impaired mitochondrial trafficking has also been reported in neuronal injury and disease, coinciding with a decrease in the number of neurites and synapses [115], [116]. Since neuronal function and viability depends on the organized transport of essential biological materials via complex mechanisms, defect in one cellular pathway might compromise other cellular functions. Mitochondria play an important role in meeting cellular energy demand, thus mitochondrial transport and distribution are crucial, particularly in the peripheral nervous system, where axons extend for great distances from the cell body.

In PD studies PINK1 has been reported to interact with Miro and Milton [117], as well as with *alpha*-Synuclein, LRRK2, and Parkin, causing disruption of the microtubule network in the cell [118]–[120]. Degenerating axons associated with alpha-synuclein containing Lewy-bodies have also been reported [121]. In [122] Liu et al. (2012) investigate the effect of PINK1 and Parkin acting to regulate Miro, a key component of the mitochondrial transport machinery. Their results indicate that altered activities of PINK1 cause abnormal mitochondrial transport. Regulation of mitochondrial transport may be a critical aspect of the mechanisms by which the PINK1/Parkin pathway participates in mitochondrial quality control. Thus dysfunction in mitochondrial transport could contribute to the loss of dopaminergic neurons.

In [123] Chu et al. (2012), examined axonal transport motor proteins throughout different stages of sporadic PD. Their findings suggested that abnormalities in axonal transport might represent a critical pathological change in sporadic and familial PD. According to [124] Abeliovich and Gitler (2016), impressive number of familial and sporadic PD genes are involved, directly or indirectly, in endosome–lysosome trafficking.

As outlined before, mutations in Mfn2 have been identified as the most common cause of CMT type 2A [69], [125], [126]. This form of CMT involves mutations in genes which are important for maintenance of axonal integrity, leading to axonal degeneration [127]. It is postulated that, either Mfn2 plays a direct role in mediating mitochondrial transport, distinct from its role in fusion, or perhaps Mfn2 mutants -induced defective fusion, may cause disruption of right mitochondrial localization, affecting the distal regions of long peripheral axons. Interestingly, recent research studies suggest that Mfn2 has an important functional relation with motor proteins that drive the axonal transport of mitochondria [110]. Experimental data from cultured neurons, showed that Mfn2 interacts directly with the Miro protein and that axonal mitochondrial transport is disrupted by loss of Mfn2 or Miro2. These findings suggest that, right axonal mitochondrial transport requires an intact Mfn2:Miro2 complex. Mutations of the Mfn2 gene have also been implicated in Hereditary Motor and Sensory Neuropathy type VI (HMSN VI), a form of CMT associated with optic atrophy [128]. This highlights the importance of mitochondrial network dynamics in optic atrophies, as well as peripheral neuropathies.

Defects in mitochondrial motility suggest deficient energy supply and altered mitochondrial dynamics, which can eventually induce neuronal dysfunction. Moreover, mitochondrial dysfunction leads to reduced ATP production levels. Since axons depend on mitochondrial production of ATP, ATP depletion, induces reduced or impaired axonal transport.

In pathologies affecting not only neurons with long axon, as in the case of AD, but also short cortex and hippocampal ones an important role for mitochondrial trafficking impairment has been identified [8], [129], [130]. In some sporadic cases of AD, mutation in Kinesin1 have been reported to produce trafficking alteration [129]. Moreover, it has been suggested that A*β* plaques may cause an effect in neuronal axons and inhibit axonal transport [131]. Both anterograde and retrograde transport is found to be altered in Amyotrophic lateral sclerosis (ALS) mouse models [132], [133]. In a HD mouse model, mutated Huntingtin protein has been observed to impair mitochondrial movement [134] and caused a redistribution of kinesin and dynein in primary cortical neurons [135].

Importantly, evidence suggests that a common characteristic of these diseases is, that axonal degeneration occurs at an early stage and may play a critical role in their pathophysiology.

## 6. Neurodegeneration and bioenergetics

Although mitochondria's shape is variable, their internal structure is highly conserved. The architecture of mitochondria comprises of two membranes, the outer and the inner. In each cycle of fusion, or fission, these membranes merge, or divide, including their contents. The outer mitochondrial membrane encompasses the inner membrane, with an intermembrane space in between. Inner membrane is the site of electron transport system and ATP synthesis. During electron transport, the participating protein complexes are ‘pumping’ protons across the mitochondrial membrane into the intermembrane space. This procedure produces a concentration gradient of protons generating chemical energy in the form of ATP.

**Table 2.** Role of mitochondrial proteins in bioenergetics.

Protein	Bioenergetic Effects	References
Mfn2	High mitochondrial membrane potential, Increased glucose oxidation, Increase in the subunits of complexes I, IV and V	[82]
Mfn2	Low mitochondrial membrane potential, Reduction of oxygen consumption, Decrease in glucose and oxidation Decrease in the subunits of complexes I, II, III and V	[82], [136]
GDAP1	Retains mitochondrial membrane potential, High mitochondrial membrane potential, Increased glucose oxidation	[85]
OPA1	Perturbations in mitochondrial membrane potential	[87], [137]

The implication of cell bioenergetics regarding the effects in mitochondrial dynamics and its correlation to diseases of the nervous system, has been reported in various studies (refer to Table 2). The interaction of mitochondrial dynamics and bioenergetics in PD has been referred in several studies [138]–[140]. More specifically, in [9], [141] Bueler, Van Laar and Berman (2009), reported that mutated alpha -synuclein and LRRK2 genes, affect, the protective/quality control role of mitochondria dynamics. Recently, in models of PD studies, results showed that, neurons preferentially enforce glycolysis as a less-efficient energy supplier, as an attempt to balance the mitochondrial dysfunction [142]. In [143] Abramov et al. (2011), study mitochondrial bioenergetics in isolated fibroblasts from PINK1 mutation patients. Their results showed different effects in mitochondria bioenergetics, including decreased mitochondrial membrane potential, changes in redox state, a respiratory deficiency and increased sensitivity to calcium overload as well as associated mitochondrial permeability pore opening.

Recent studies, have shown that mitochondria are significantly deteriorated in AD supporting both a spatial and temporal relation between increase neuronal oxidative damage and mitochondrial abnormalities [91]. In [8], Wang et al. (2009), reported that in APP overexpressing cells mitochondria's fusion rate was much slower. Moreover they detected mitochondrial fragmentation in differentiated primary hippocampal neurons and also an increase in ROS production, reduced ATP generation and depression of mitochondrial membrane potential, characteristics of impaired mitochondrial function [95].

In autosomal-recessive CMT type4A patients' fibroblasts, Noack et al. (2012) in [85] detected a decline in GDAP1 levels and also a decreased mitochondrial membrane potential. Their experiments with cultured cells indicated that GDAP1 gains cellular GSH content in neuronal cells, while deteriorates ROS production, possibly due to balanced mitochondrial membrane potential and respiratory efficacy, acting conservatively against oxidative stress. Other research studies identified an independent role of Mfn2, apart from its role in mitochondrial fusion. Experimental data using muscle cells, indicated that Mfn2 alters mitochondrial metabolism and its expression level controls mitochondrial membrane potential and the oxidative phosporylation (OXPHOS) system through fuel oxidation [82], (refer to Table 2). Pinch et al. (2005) postulated that this manifestation may justify the delicate sensitivity of neuronal cells to Mfn2 loss-of function, described in cases of CMT2A. In another study with fibroblasts from CMT2A patients, researchers reported defect of mitochondrial coupling, associated with defects in membrane potential and decreased respiratory capacity of mitochondria [128]. Moreover, in other studies researchers concluded that the underlying mechanisms of disease-causing dominant Mfn2 mutations might be alterations of the physiology of mitochondria [84].

## 7. Discussion

**Figure 2.**
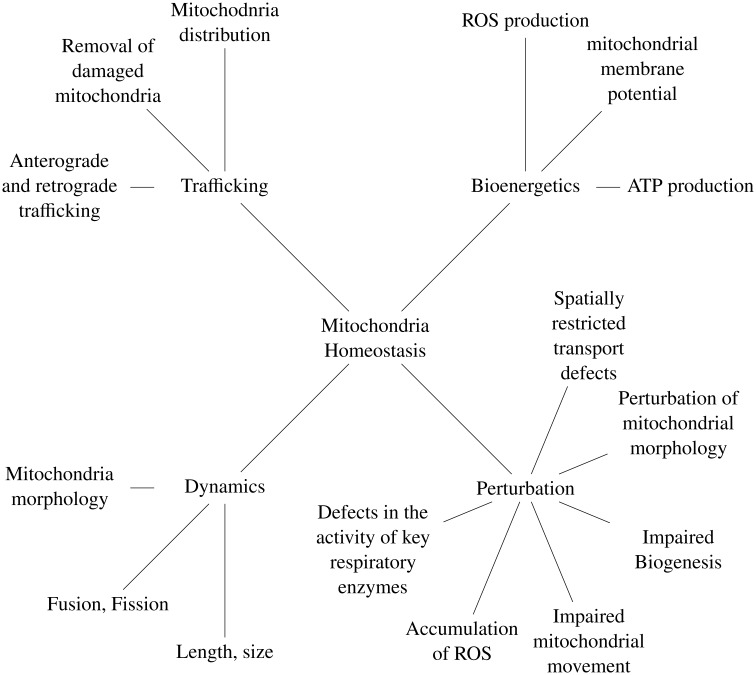
Mitochondria homeostasis and perturbation in neuronal diseases.

Three aspects of mitochondria's function, dynamics, trafficking and bioenergetics, are fundamental in mitochondrial homeostasis, while perturbation in the quality control of this characteristic is implicated in neuronal diseases (refer to Figure 2).

Whether mitochondrial dysfunction is a cause, or consequence of the pathogenesis of the disease, remains to be fully elucidated. Since there is growing evidence, that impaired mitochondrial function has an important role in the pathogenic mechanisms of neurodegeneration, it is interesting to concentrate on developing methods to repair damaged mitochondrial function, or to use mitochondrial evolution as a diagnostic procedure. The problem with all of the individual studies, related to a complex human disease, is that the research question have been focused to a single phenomenon which is measurable or testable; for example, “fibrillar protein deposits cause neurodegenerative disease” [144]. In order to further define the research question in such a complicated problem, it is crucial to examine the dynamic changes in mitochondrial structure associated with disorders in mitochondrial function and energy state. Apparently, there is number of (un)correlated factors which contribute to mitochondrial network and morphology. These biological pathways which regulate the shape and function of mitochondria are linked to a number of neurodegenerative disorders. Evidently, due to the intricate mechanism of neurodegenerative diseases, it is difficult to determine in the laboratory environment the direct effects of mitochondrial shape, number, and location to the development of the disease.

For example, in case of post-mortem studies in individuals with PD, it is evident that mitochondrial defect is sufficient to cause loss of substantia nigra (SN) neurons. Over the past years, the growing understanding of the molecular pathways involved in the development of PD, has identified a role for mitochondrial dysfunction, in terms of ATP and calcium buffering capacity and also dynamics, transport and distribution. Since different aspects of mitochondria defects have been implicated in molecular genetic pathways in PD (reviewed in [145], [146]) it is difficult to unravel “cause and effect” in the contribution of mitochondrial dysfunction to the pathogenesis of the disease. Genetic techniques have enabled researchers to identify disease-causing genes in PD, associated to mitochondrial disorders. These changes in mitochondrial pathways, activate responses, to regulate mitochondrial and cellular homeostasis. It is suggested that neurons become more sensitive due to these responses, ultimately leading to degeneration and impaired function [147]. Further evidence, support that a decline in mitophagy, is an important component of ageing [148]. While several studies entangle impaired mitochondrial turnover in neuronal loss in PD [149], [150], accumulation of alpha-synuclein in neurons, even in idiopathic PD, may put an additional burden in the regular protein turnover and the removal of altered proteins, increasing the vulnerability of SN neurons to mitochondrial dysfunction [151]. Furthermore, many of the genes responsible for familial PD, have been shown to have important roles in autophagy [61], [152], [153], a process which is essential for neuronal survival. Thus, its dysregulation is connected to neurodegeneration. Together, these data suggest that many of the proteins that are known to play a casual role to the molecular mechanisms underlying PD, have also been linked to dysfunction in dynamics, distribution, and transport of mitochondria within neurons.

It is clear that the correct shape and distribution of mitochondria within neurons is important to the proper function of these organelles, as well as to brain energetics. While current techniques and models of the molecular signaling pathways involved in PD, have been developed within the context of mitochondrial homeostasis, the complex nature of mitochondria establishes an intricate link of mitochondrial dynamics, turnover, quality control and trafficking in cases of PD.

While modelling complex biological phenomena, it is essential to discern the nature of the biological process in order to choose the appropriate components of the modelling method. Constructing a model includes the derived methods, analogies and context, in order to represent the mechanisms by which biologists describe the behaviors of complex molecular and cellular systems. Validation of these models depends on systematic verification of data published in literature. Since the experiments are usually conducted *in vitro*, describing complicated systems, often requires to make assumptions in order to use simpler mathematical models. Considering the need to model such a composite biological system both in spatial and temporal level, there is a challenge to use a formal modeling framework which describes both a qualitative and a quantitative structure of mitochondrial homeostasis. Formal models offer a dynamic framework of modeling, which allows to analyze various parameters integrated in a time-dependent system and has been used for modeling biological networks at multiple scales [154]–[158].

As discussed previously, to preserve the integrity of mitochondrial population is critical in order to maintain cell health and disruption of that integrity is strongly linked to neurodegenerative diseases [159]. Homeostasis of mitochondrial structural network is influenced by a critical regulation of fission and fusion. As clearly stated in literature experimental studies, a number of components that govern the fission and fusion machinery, including Drp1, Mfn1/Mfn2 and OPA1, are involved in mitochondrial shape changes and influence the process of autophagy and apoptosis. Impaired coordination between these processes appears as a key feature of several neurodegenerative and neuromuscular disorders. Researchers have identified a direct molecular link between mitochondria and the pathogenesis of hereditary early-onset PD. More specifically, mutations in PINK1 and Parkin have been reported in autosomal recessive PD [75], [160]. Recently, PD-familial related genes, such as alpha-synuclein, Parkin, PINK1, DJ-1 and LRRK2, have been identified in mitochondria dynamics, as moderators of mitochondrial integrity and function [57]. Mutations in LRRK2 have been reported as a common cause of familial and sporadic PD [161]. Furthermore, it is not only the form of functional mitochondria which needs to be monitored and controlled, but also their localisation and transport.

Although it is difficult to understand mitochondria dynamics from *in vivo* studies, computational techniques provide an alternative to interpret and make sense of the patterns and changes in this complex network [162]–[164]. Depending upon the particular question being asked, simulation of mitochondrial network using various modeling methods, is performed, in order experimental quantitative knowledge of parameters to be extracted. In [165] Shah et al. (2019) examine the changes in the fission and fusion processes, due to lateral and longitudinal interactions in neurodegenerative pathologies. They used images of mitochondrial networks in cells with various neurological pathologies including AD, ALS, PD and other and found significant differences between their microscopic properties. From these findings, concluded that, microscopic differences may lead to major imbalance in mitochondria structure and topology, which may have significant implications in various cell functions in different diseases.

In another study of mitochondrial dysfunction, Toglia et al. (2018) combined multiscale modeling, electrophysiology and fluorescence imaging techniques, to show that exogenously applied intracellular A*β* oligomers leads to impaired mitochondrial function [166]. This study combines key observations in different levels in order to reproduce a multiscale model, that allows to estimate quantitave information of various components involved in the *Ca*^2+^ signaling disruption and its downstream effects.

Computational models are playing an ever increasing role in the past twenty years and serve scientists to give solutions to important questions, which are significant in biology. In conclusion, future studies should address to the challenging problem of unveiling the regulatory factors for mitochondrial dynamics and how they associate to quality control. Computational modelling of mitochondrial biology is of particular interest, since targets aimed at restoring mitochodnrial dynamics have emerged as therapeutic strategies with broad application across health.

## 8. Conclusion

It has been hypothesized that the pathogenic processes connected with neurodegeneration in PD and AD start decades before the first symptoms. The use of treatments that slow down disease progression would have a significant effect, if disease can be early diagnosed and treated presymptomatically. In recent studies, live imaging of mitochondrial dynamics in CNS dopaminergic neurons in vivo supports a potential link of mitochondrial dynamics in early PD neuropathology [167]. Recently the diagnostic criteria of AD have been revised to include brain imaging and cerebrospinal fluid (CSF) biomarkers, in order to evaluate the likelihood a patient has an ongoing AD pathology or not. These biomarkers may identify early stages of the disease progression.

Over the last years many research studies have focused on the reduction of both A*β* formation and aggregation, as potential therapeutic strategies [168]–[170]. Still, there are researches which are exploring whether protein aggregates are a cause, or a consequence of neurodegenerative diseases such as AD.

Emerging evidence show that amyloid precursor protein (APP), increases the production of A*β* and may lead to a lack of balance in mitochondrial fission/fusion. Studies results suggest that mitochondrial fragmentation and abnormal distribution, may be implicated in mitochondrial and neuronal dysfunction [95]. Precedent exists which proposes the relevance of mitochondria's fusion and fission pathways, to alterations in energy metabolism and vice-versa. The extent of synaptic loss in the brain is associated with cognitive impairment in clinical AD. Since the degree of synaptic dysfunction correlates with impaired mitochondrial function in the nerve ending, this is a possible therapeutic target. More accurate determination of the regulatory mechanisms of robust mitochondrial population is essential for early detection of neurodegenerative diseases. Critical regulation of mitochondrial distribution and transport is an important step towards understanding underlying pathways leading to neuronal dysfunction. Some researchers have proposed that the mechanisms of directional transport regulation should include a mitochondrial sensor for membrane potential, the flux, or concentration of a metabolite or ion (H^+^, Ca^2+^), ATP and ADP levels [171]–[173]. Others, speculate that neuronal survival may be promoted by redistribution, fission and autophagy, mechanisms, which increase bioenergetics efficiency [11], [174], [175]. It will be of critical interest to distinguish compensatory and pathogenic changes, in order to identify possible therapeutic targets. Manipulation of mitochondria's quality control represents a reasonable therapeutic approach for treatment of neurodegeneration.
